# The influence of UV radiation on the properties of GFRP laminates in underwater conditions

**DOI:** 10.1038/s41598-024-57999-8

**Published:** 2024-03-28

**Authors:** Jakub Smoleń, Piotr Olesik, Bartłomiej Nowacki, Marcin Godzierz, Klaudia Kurtyka, Paweł Chaber, Jan Czakiert, Mateusz Kozioł

**Affiliations:** 1 Faculty of Materials Engineering, Silesian University of Technology, Krasińskiego 8 Street, 40-019 Katowice, Poland; 2 Faculty of Materials Science, Department of Industrial Informatics, Joint Doctoral School, Silesian University of Technology, Krasińskiego 8, 40-019 Katowice, Poland; 3 Centre of Polymer and Carbon Materials, Polish Academy of Sciences, M. Curie-Skłodowskiej 34 Street, 41-819 Zabrze, Poland; 4 DeGroote School of Business, McMaster University, 1280 Main St W, Hamilton, ON L8S 4L8 Canada

**Keywords:** Polymer matrix composites, UV degradation, Environmental degradation, Unsaturated polyester resin, Glass fibers, GFRP laminates, Materials science, Techniques and instrumentation

## Abstract

Degradation of polymer composites is a significant problem in many engineering aspects. Due to the interaction of various degradation factors during the exploitation of composites, a synergistic effect of destruction is observed. The article describes the phenomena occurring in glass fiber reinforced polyester laminates under the influence of ultraviolet radiation (UV) in an aquatic environment. The laminates were exposed to UV-A, UV-B and UV-C radiation for 1000 h in free-air and underwater conditions. During the test, the materials were immersed at stable depth of 1 mm and 10 mm, respectively. The three-point bending tests performed on the samples after being exposed to UV showed an increase in the flexural strength of the composites. Simultaneously, degradation of the outer surface layer was observed. The degradation removed the thin resin film from the surface which resulted in a direct exposure of the reinforcing fibers to the environment. The transformations taking place in the deeper layers of the composite increased the mechanical strength due to the additional cross-linking reactions excited by the energy arising from the radiation. Moreover, the formation of polymer structures from free styrene remaining after the technological process and the occurrence of free radical reactions as a result of the cage effect was also observed.

## Introduction

The complex nature of solar radiation exerts a varied influence on both organic and inorganic matter. The solar radiation includes visible radiation (wavelength range from 400 to 700 nm)^1,2^, however also includes ultraviolet radiation, such as UV-A (315–400 nm), UV-B (280–315 nm) and UV-C (100–280 nm) as well as infrared radiation^3,4^. The effect of solar radiation is noticeable and can have a positive or negative impact in many fields. The positive aspects of radiation include the synthesis of vitamin D in the human body, while the negative effect of the interaction is a carcinogenic process. Similar effects are observed in materials, especially polymeric materials.

**Figure 1:**
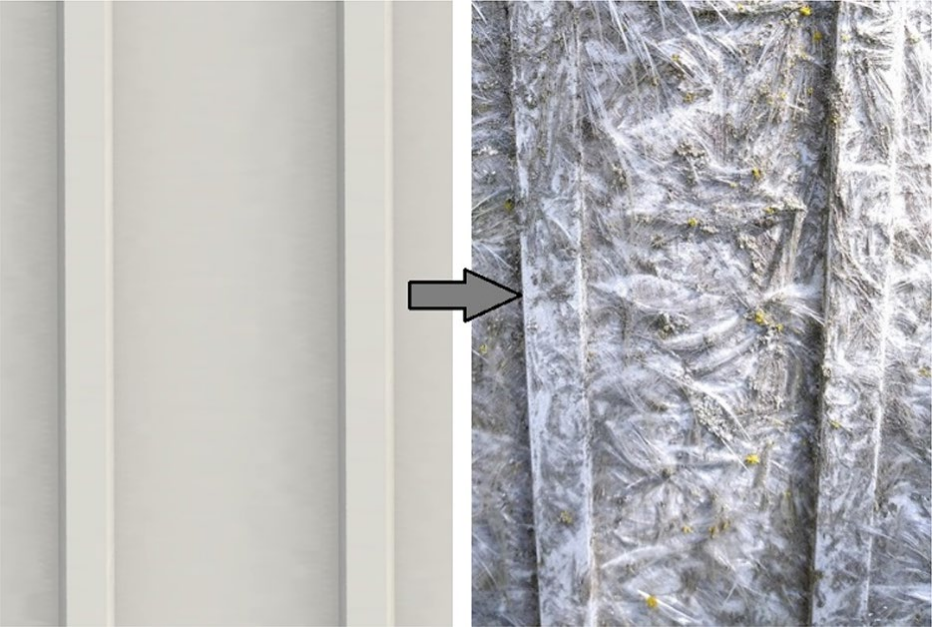
Degradation of the fiber-reinforced polymer composite after ten years of environmental exposure (electrical device housing).

One of the biggest disadvantages of polymers is their low resistance to solar radiation, including UV radiation. The degradation of polymer products is manifested by their brittleness, loss or change of color and tarnishing. In addition to its change in appearance, the material also undergoes internal changes in chemical and physical properties. Changes in the appearance of the material are closely related to internal structural changes that occur in the polymer structure as a result of degradation. The response of the polymer material to absorbed UV radiation is to break molecular bonds or create new chemical groups. The yellowing typical of polymers results from the formation of chromophore groups in the material, which, by absorbing visible light radiation, may contribute to a change in color. Breaking chemical bonds often contributes to loss of elasticity, increased brittleness, deterioration of mechanical strength, decreased impact strength and elastic modulus. Structural changes also lead to a change in the degree of crystallinity of the polymer, which translates into changes in properties^5,6^. Polymers and polymer-matrix composites degrade under the influence of various factors, among which the most common types of degradation include thermal^7–10^, thermal-oxidative^11^, chemical^12–14^, mechanical^15–18^, hydrolytic^19,20^, photodegradation^21–25^ and biodegradation^26,27^. During use in normal conditions, the products undergo complex degradation caused by more than one factor. The complexity of the phenomena affecting the destruction of the material often has a synergistic effect, which additionally accelerates its destructive processes. An example of the synergistic interaction of several degradation types (including thermal, hydrolytic, photodegradation and biodegradation) can be the destruction of electrical equipment housings made of fiber-reinforced polymer composites, as shown in Fig. 1.

The resistance to photodegradation of polymeric materials depends on the structure, additives, technological processes, and the presence of impurities in the material^28^. Polymer photodegradation occurs when the absorbed quantum of UV radiation destroys the bonds of the chromophore photosensitive molecule. According to Latos et al.^29^, photodegradation of polymeric materials includes processes that lead to a reduction in molar mass, but also processes that lead to cross-linking and formation of branched structures. In addition, UV radiation shows a surface effect, and the products of oxidative photodegradation are observed in a thin, subsurface layer of the material with a depth of about 40 µm^30^. The catalyzing effect of oxygen in the photodegradation process causes the material to undergo photooxidation. Despite the initial surface nature of the interaction of radiation with the material, oxygen atoms diffuse into deeper areas of the material, which then directly contributes to the degradation of the entire polymer volume. Oxygen diffusion is easier and occurs faster in the amorphous structure than in the crystalline phase of polymers, where oxygen transfer is difficult due to the dense structural network^31^. Therefore, it is beneficial to protect amorphous polymers with layers of crystalline polymers, as is the case in the production of polycarbonate plates with an amorphous structure covered with a thin layer of acrylic, protecting the material against photodegradation^32^.

Water is a photochemically inactive solvent, but it may be involved in promoting the photodegradation of polymers due to hydrolysis reactions. However, an aquatic environment where photodegradation is taking place can lead to the “cage effect”. As a result of free radicals being trapped at the point of their formation, they undergo recombination causing an undesirable reaction with a side group, possibly delaying the initiation of the material's degradation^33–36^. During operation, laminates are subjected to mechanical stress. The weakening of composite materials in some areas leads to the formation of cracks and even delamination, which is the main disadvantage of this material^37,38^. The resulting microcracks and other discontinuities in the material increase the surface of the material's interaction with water and facilitate the diffusion of atmospheric oxygen into the material structure, which additionally accelerates the degradation processes. The diffusion of water into the polymer structure is closely related to its hydrophilic nature, temperature and the presence of ions in the aqueous environment. Water ingress leads to gradual plasticization of the polymer and hydroscopic swelling, which affects the mobility of the polymer chains while increasing mechanical stress and leading to further stretching of the polymer chains. The mobility of polymer chains in the solid state is low, and the rate of photochemical reactions depends on the rate of diffusion of low-molecular active products, such as oxygen. The cage effect occurring in photoreactions may be limited by the hydrophilic nature of the polymer, which increases the mobility of the polymer chains. However, in the case of hydrophobic polymers, water diffusion may reduce chain mobility, which may ultimately inhibit photodegradation through recombinations and reactions of free radicals with the polymer structure^39–41^.

The number of scientific works dedicated to the description of photodegradation and environmental degradation of polymers is large. Although the degradation mechanisms in various polymers have been known and described, the literature describing the phenomena occurring in laminates is rare. The behavior of the laminate differs from unfilled polymer matrix material, as there are numerous interactions between the matrix and the reinforcement of the composite, which have a complex impact on the final effect. It is therefore advantageous to describe the photodegradation under UV radiation in laminates. The multiphase structure of fiber-reinforced composites is characterized by the complex nature of phenomena occurring as a result of chemical degradation, including hydrolysis. The nature of the hydrolysis of the reinforcement is different from the hydrolysis of the matrix material and interphase. Hydroscopic swelling of the polymer matrix contributes to the plasticization of the structure and increased chain mobility, which generates an increase in mechanical stress. Hydroscope swelling in GFRP composites is anisotropic in nature, where swelling in the direction of the fibers is restrained by the unswellable glass fibers, and swelling along the fibers is free in nature, similar to the case of an unconstrained polymer. Changes in internal stresses occurring as a result of water absorption may lead to fatigue damage in the interlayer and intralayer regions of composites^42–44^. This research work covers the UV radiation testing of glass fiber (plain woven fabric) reinforced polyester laminate in an aqueous environment. In order to describe the effect of UV radiation, the samples were exposed to UV-A, UV-B and UV-C radiation in dry conditions and in constant water immersion at depth of 1 mm and 10 mm for 1000 h. Description of the phenomena of photodegradation of the FRP composite laminates in UV-A and UV-B radiation (in both aquatic and dry environments) is essential in future development of applications such as boats, wind turbines, bridge components, and pipes. UV-C radiation, absorbed entirely by the Earth's atmosphere, does not pose a threat to the durability of the materials under normal operating conditions. However, UV-C radiation is commonly used in disinfecting installations, e.g. water purification installations and installations for disinfecting rooms and surfaces, which was particularly important during the SARS-CoV-2 pandemic^45–48^. The UV-C radiation is also present in outer space. The study of the phenomena occurring in polyester-glass laminates is important for extending the lifetime of products and protecting human health and life.

## Materials and methods

### Designing the UV test system

#### Construction of the UV chamber

The planned studies of photoaging processes in non-standard conditions made it necessary to design and build a dedicated research UV-exposure system. The system includes four separate test zones, including the radiation type: UV-A, UV-B, UV-C, and the zone without radiation (“dark zone”). The zones are separated by partitions that exclude the possibility of radiation penetrating from one zone to the other. Each test zone has 300 × 200 × 200 mm containers filled with distilled water (pH close to 7.0). There was one separate water container for one sample. The separateness of the containers excluded the possibility of mixing photodegradation products between the series of samples. Glass was selected as the most advantageous material for the containers because it is a chemically inert material and does not undergo photodegradation. The water level was kept constant throughout the test. Composite samples were immersed in water with a constant depth of 1 mm and 10 mm, respectively.

**Figure 2:**
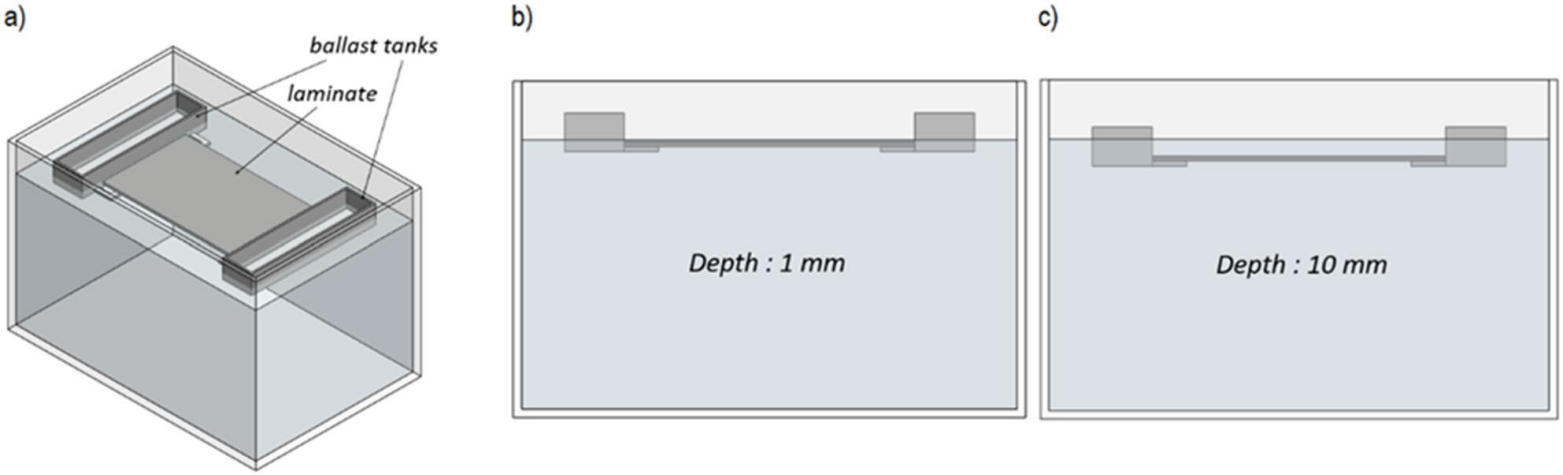
(**a**) sample of polyester-glass laminate attached to ballast tanks, (**b**) sample immersed to a depth of 1 mm, (**c**) sample immersed to a depth of 10 mm.

Considering slight deviations in the water level in the container due to evaporation, a ballast system was designed, ensuring constant immersion of the samples, regardless of changes in the water volume remaining in the container. Ballast tanks were designed and attached to the sample during the test. Changing the ballast tank load makes it possible to vary the draft of the sample. The ballast tank material must not be water-absorbent, because it would drown the specimen and cause the test to fail. They also represent a specific, complex shape. Therefore, the ballast tanks were manufactured from polylactide (PLA)—polymeric material resistant to water absorption and UV-stable for over 1000 h, using the additive printing technique (FDM). Quartz sand was used to load the ballast tanks. The sample attached to the ballast tanks is shown in Fig. 2.

#### Selection of UV lamps

It was assumed that all the lamps used in the tests have the same power [W] and have the same shape and dimensions. The necessary condition was a narrow wavelength range. The spectral power distribution for UV-A lamps is the wavelength range from 350 to 400 nm (Philips PL-L 36W/10), for UV-B lamps 311 nm (Philips PL-L 36W/01) and UV-C lamps 254 nm (Philips TUV PL-L 36W/4). Lamps were mounted horizontally, parallel to the plane of the sample. The distance between the lamp and the sample was 100 mm + / − 5 mm (depending on the changes in the height of the water in the container due to evaporation and refilling).

**Figure 3:**
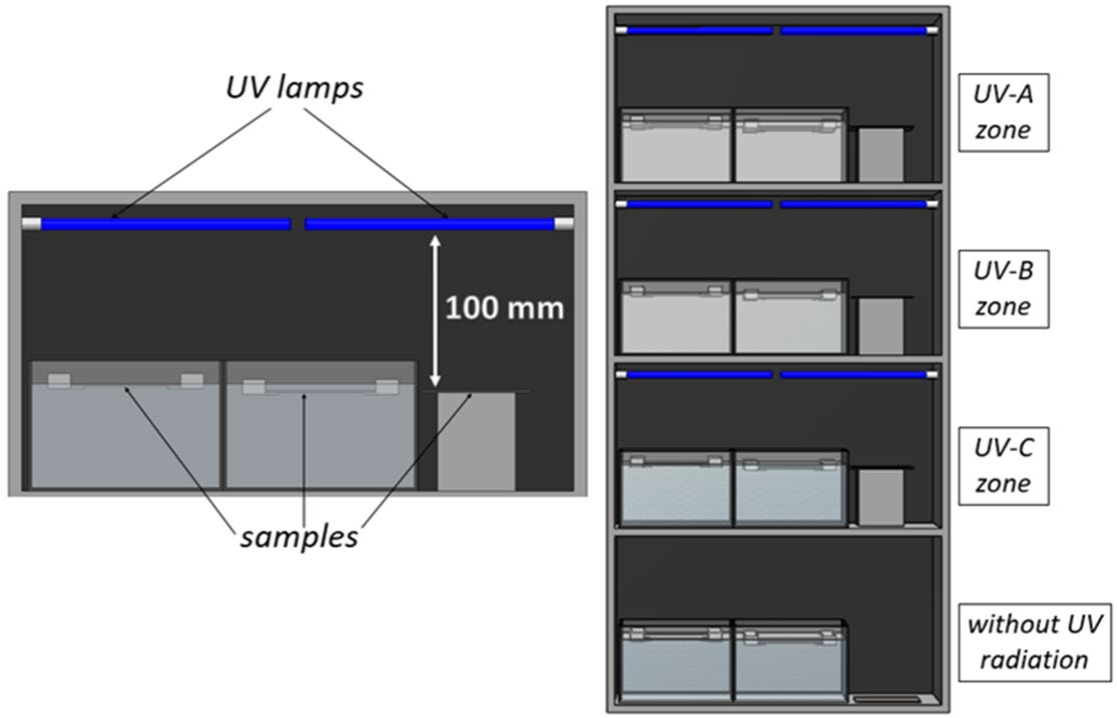
Graphical visualization of the UV chamber used to test samples with separate radiation zones: UV-A, UV-B, UV-C and a zone without radiation.

Figure 3 shows a schematic view of the completed UV chamber with four test zones. Each level corresponds to a different radiation zone. On one level there is a container with a sample immersed to a depth of 1 mm, a container with a sample immersed to a depth of 10 mm, and a container with the sample tested in dry conditions. The dark zone is the lowest level of the UV chamber, interpreted as the reference zone.

### Preparation of samples

**Figure 4:**
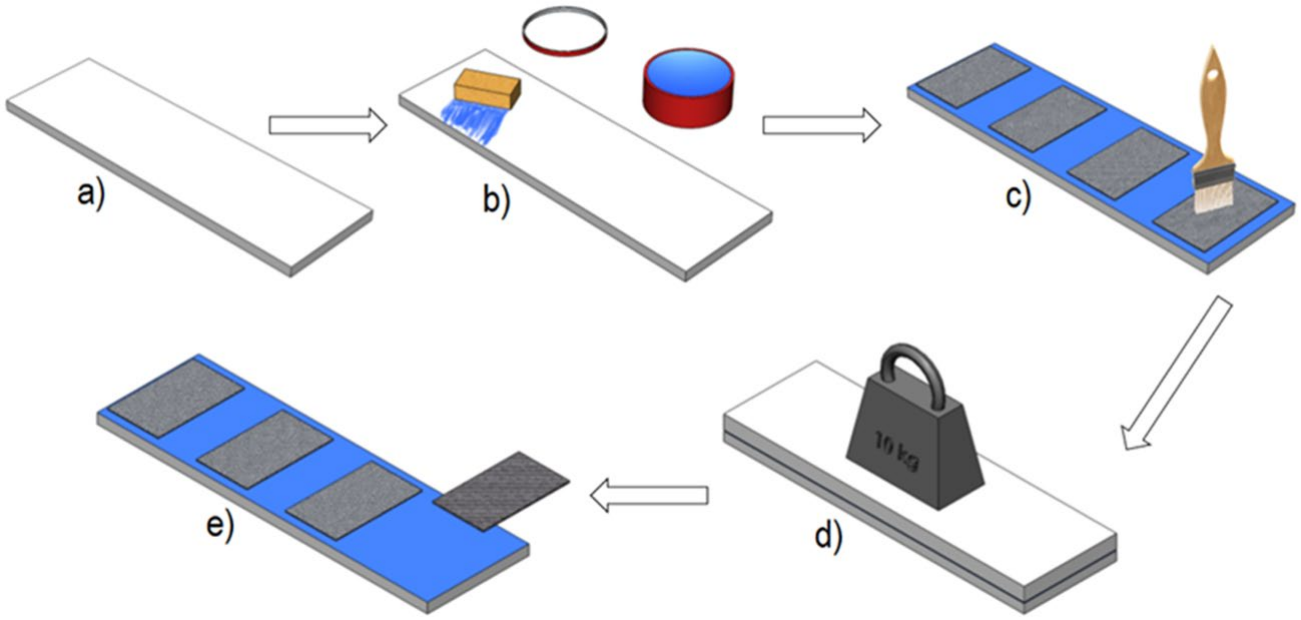
Scheme of the manufacturing of polyester-glass laminate with hand lay-up technique. (**a**) preparation of materials, (**b**) application of a release agent—wax, (**c**) production of a 6-layer laminates, (**d**) mold closure and loading, (**e**) demolding of cured composites and post-curing process.

The matrix of studied laminates was HAVELPOL-1 orthophthalic unsaturated polyester resin (Havel Composites, Czech Republic) catalyzed with methyl ethyl ketone peroxide (MEKP) (Butanox M50, AkzoNobel, the Netherlands). The weight ratio of resin and catalyst was 100: 2. The sample production scheme is shown in Fig. 4. In the first stage, preforms with dimensions of 170 × 100 mm were assembled from a plain-woven glass fabric (320 g/m^2^, KROSSGLASS, Poland).

A layer of release wax was applied to the surface of the flat plate. Laminates consisting of six layers of glass fabric were made using the hand lay-up technique. In the laminate production process, a loading plate (10 kg in weight) was used to obtain an even thickness of the laminates and a smooth composite surface. The pressure exerted on the surface of the laminate was roughly 1 kPa. The use of the loading plate made it possible to remove excess resin from the structure of the material. After 24 h, the composites were demolded and post-cured for 12 h at the temperature of 60 °C. After the sample preparation process was completed, the excess resin was cut off from the edges of the samples, and the samples were attached to the ballast tanks. For the purposes of the research, twelve laminate samples were produced and subjected to UV irradiation 14 days after their production. The samples were marked as described in Table 1.

**Table 1:** Sample designation within the study.

	Immersion depth 0 mm	Immersion depth 1 mm	Immersion depth 10 mm
Without radiation	P.0	P.1	P.10
UV-A radiation	P.UVA.0	P. UVA.1	P. UVA.10
UV-B radiation	P.UVB.0	P. UVB.1	P. UVB.10
UV-C radiation	P. UVC.0	P. UVC.1	P. UVC.10

### Testing procedure

#### Surface visual inspection

The surface of the GFRP laminates was visually inspected using an OLYMPUS DSX1000 digital optical microscope (Olympus, Tokyo, Japan). Using the dark field (DF) technique, an image of the surface of the samples was recorded at 1 × magnification in the area of 10 × 10 mm. In addition, a high-resolution 3D visualization was performed to help describe changes in the surface profile. Initial observation of the entire surface of the GFRP composite allowed us to conclude that there are no areas on the surface that differ in appearance. For all samples, areas were selected in the central area of the composite. 2D images and 3D visualization were made in the same area. 3D visualization was performed for 12 parallels to obtain high detail of the images.

#### Mechanical properties

A set of 100 × 20 mm samples for the static three-point bending test was cut out of the GFRP laminates. The test was carried out in accordance with PN-EN ISO 14,125 on a Shimadzu AGX-V machine (Japan). Flexural strength [MPa], Young's modulus [GPa] and maximum deflection [%] at 23 °C were determined. The spacing distances between supports was 60 mm and the strain rate was 10 mm/min. The obtained results were evaluated with the Grubbs test for significant outliers. Insufficient results were discarded, and the means were statistically compared by ANOVA followed by a post-hoc Tukey test.

#### FTIR spectroscopy

In order to describe the internal changes occurring in the structure of the GFRP composite after UV exposure, FTIR spectroscopy was performed. A Thermo Scientific Nicolet 6700 spectrometer with attenuated total reflection (ATR) was used. For this purpose, Smart Orbit with a diamond crystal was used. The intensities of the spectra were normalized to the band 1377 cm^−1^, derived from the vibrations of the CH_3_ groups.

## Results and discussion

### Evaluation of surface changes

**Figure 5:**
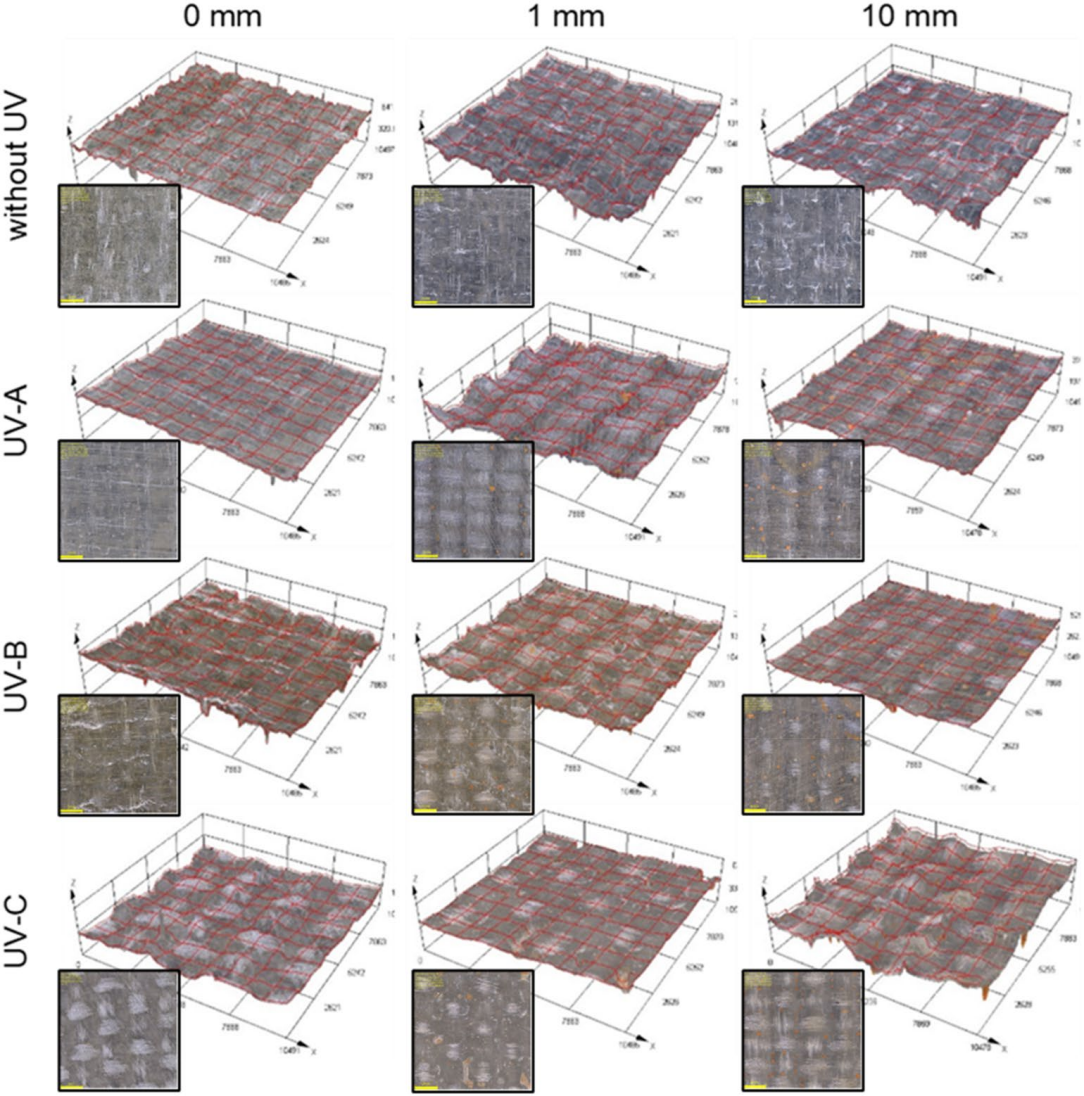
The surface of polyester-glass laminates after 1000 h (Olympus DSX1000, Japan).

**Figure 6:**
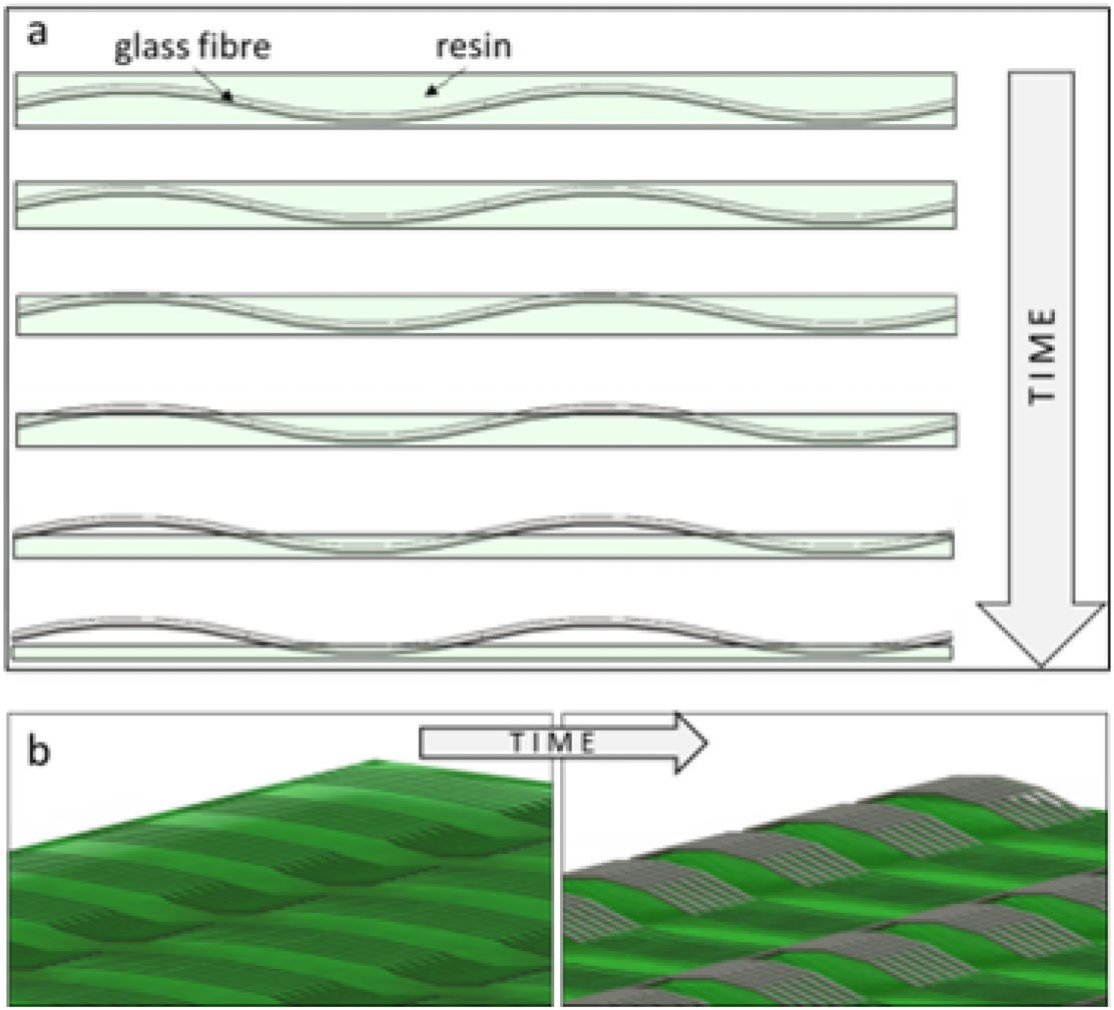
Visualization of changes occurring on the surface of the GFRP laminate during photodegradation (**a**) change in thickness over time, (**b**) exposure of fibers on the surface.

The surface of the composite (Fig. 5) undergoes significant changes during photoaging. A color change of the sample from light green to brown is observed. The reference sample (P.0) is characterized by a smooth surface without protruding glass fibers. Immersion of samples in water does not lead to changes in the surface of the laminate, but a color change is observed partly due to hydrolysis. Ultraviolet radiation causes a change in color and surface profile. The change in the surface of the GFRP laminate is large and exposes the glass fibers on the surface due to the loss of polyester resin (Fig. 6b). Visualization of the ongoing degradation process over time is shown in Fig. 6a. Protruding glass fibers are dangerous for users and can lead to injury of human skin. The greatest surface changes were observed in the samples exposed to UV-B and UV-C. Underwater immersion only partially protects the laminate surface from photodegradation, which is evidenced by a smaller color change between the sample immersed at a depth of 10 mm and 1 mm, but the hydrolysis processes are intensified, which contributes to the diverse nature of surface defects. The samples with the highest amount of glass fibers on the surface are the following samples: UV-A (1 mm), UV-B (0 mm), UV-B (1 mm), UV-C (0 mm), UV-C (10 mm).

### Evaluation of changes in mechanical performance

**Figure 7:**
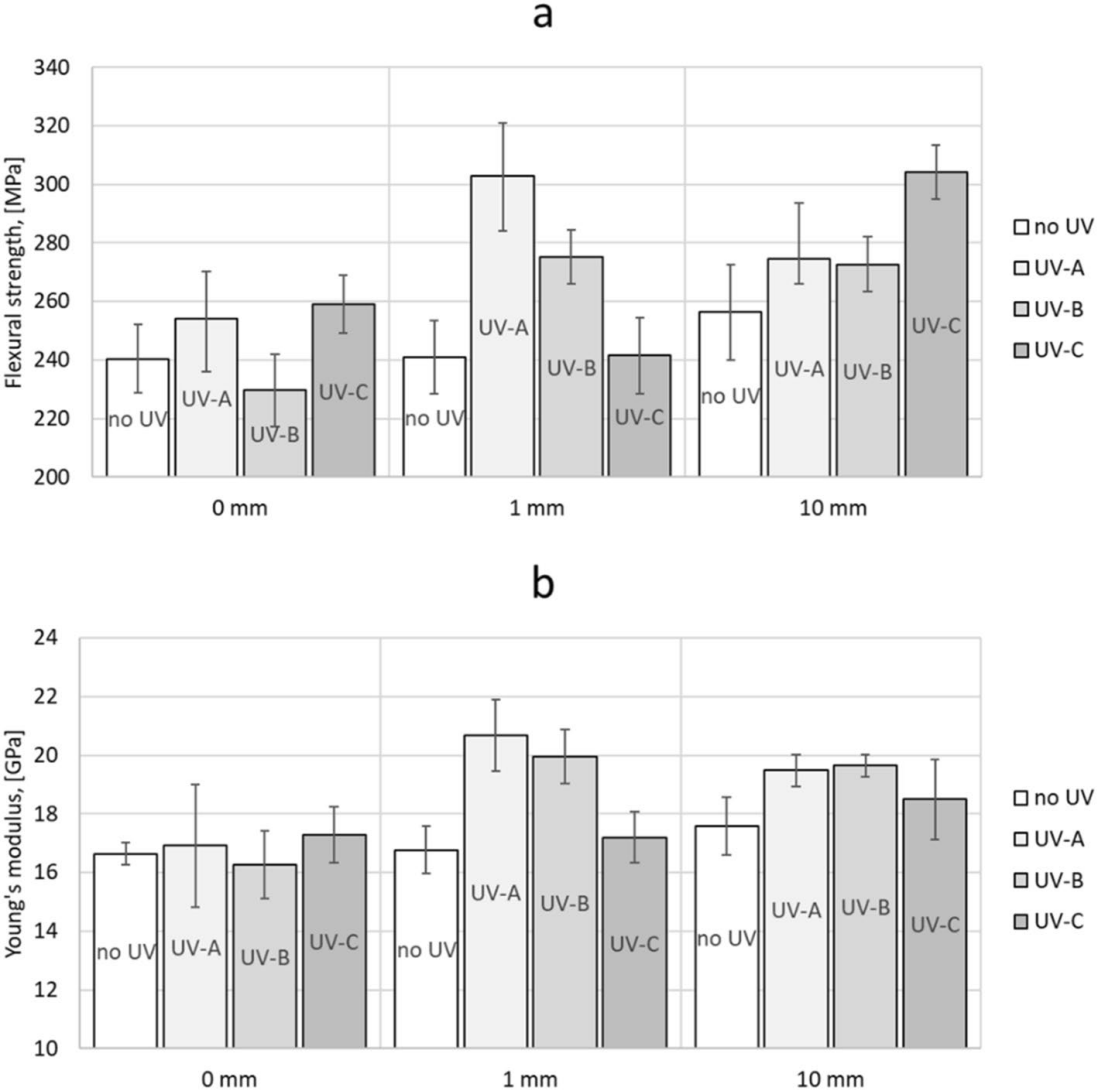
Average values of flexural strength [MPa] (**a**), Young’s modulus [GPa] (**b**).

A three-point bending test was carried out and the test results are shown in Fig. 7 and Table 2. The obtained results were subjected to statistical analysis in order to indicate significant differences in values. Reference samples (without access to UV radiation) do not show large changes in mechanical properties depending on access to water or not. The bending strength of the reference GFRP laminate is about 240 MPa. Young's modulus does not change significantly in samples without access to ultraviolet radiation (Table 2).

**Table 2:** Three-point bending test results.

Sample	Flexural strength, [MPa]	Maximum deflection, [%]	Young's modulus, [GPa]
P.0*	240.42 ± 11.59	2.09 ± 0.23	16.63 ± 0.38
P.UVA.0	253.95 ± 18.10	1.94 ± 0.32	16.91 ± 2.10
P.UVB.0	229.68 ± 12.30	2.03 ± 0.19	16.27 ± 1.16
P.UVC.0	258.94 ± 9.92	1.83 ± 0.13	17.28 ± 0.96
P.1*	240.89 ± 12.57	2.00 ± 0.21	16.76 ± 0.81
P.UVA.1	302.95 ± 18.93	1.82 ± 0.11	20.69 ± 1.22
P.UVB.1	275.25 ± 9.16	1.69 ± 0.06	19.96 ± 0.92
P.UVC.1	241.48 ± 13.08	1.96 ± 0.30	17.20 ± 0.88
P.10*	256.25 ± 16.44	1.70 ± 0.06	17.59 ± 0.99
P.UVA.10	274.59 ± 8.77	1.96 ± 0.33	19.48 ± 0.54
P.UVB.10	272.70 ± 9.50	2.02 ± 0.32	19.65 ± 0.38
P.UVC.10	304.23 ± 9.31	2.00 ± 0.11	18.50 ± 1.36

*Samples not exposed to ultraviolet radiation (dark zone).

UV-A radiation leads to a significant change in the mechanical properties of the non-immersed sample (P.UVA.0), for which the flexural strength is about 254 MPa and the Young's modulus is 16.91 GPa. The GFRP laminate immersed to a depth of 1 mm is characterized by a significant increase in bending strength (about 303 MPa), and an increase in Young's modulus (20.69 GPa). Reducing the immersion depth in water to 10 mm leads to small changes in mechanical properties, which suggests that the radiation energy in the UV-A wavelength range decreases with increasing immersion depth in water.

UV-B radiation is particularly unfavorable for the non-immersed sample (P.UVB.0), where the bending strength decreases (about 230 MPa), but the Young's modulus does not change. Samples immersed in water, regardless of the depth, undergo a similar change in bending strength, where about 275 MPa (for the P.UVB.1 sample) and about 273 MPa (for the P.UVB.10 sample) were reached. Both samples are characterized by a significant increase in mechanical strength, while also in the range of Young's modulus (about 20 GPa). UV-B radiation is characterized by higher energy than UV-A radiation. Due to this, water with a depth of 10 mm does not provide more protection for the material than water with a depth of 1 mm.

UV-C radiation also leads to changes in the strength of the material. For the non-immersed sample (P.UVC.0) and the one immersed to a depth of 1 mm (P.UVC.1), a similar, slight increase in flexural strength was recorded compared to the reference sample (P.0), 259 MPa and 241 MPa, respectively. There is no significant change in Young's modulus in these laminates. The greatest change in bending strength was observed in the sample immersed to a depth of 10 mm (P.UVC.10), where a value of 304 MPa was reached and a significant increase in Young's modulus to 18.5 GPa was detected. Water with a depth of 10 mm is not a barrier to high-energy UV-C radiation. The large difference in values between immersion to a depth of 1 mm and 10 mm proves a strong, synergistic effect of the interaction of UV-C radiation and water.

### Chemical explanation of the observed changes

**Figure 8:**
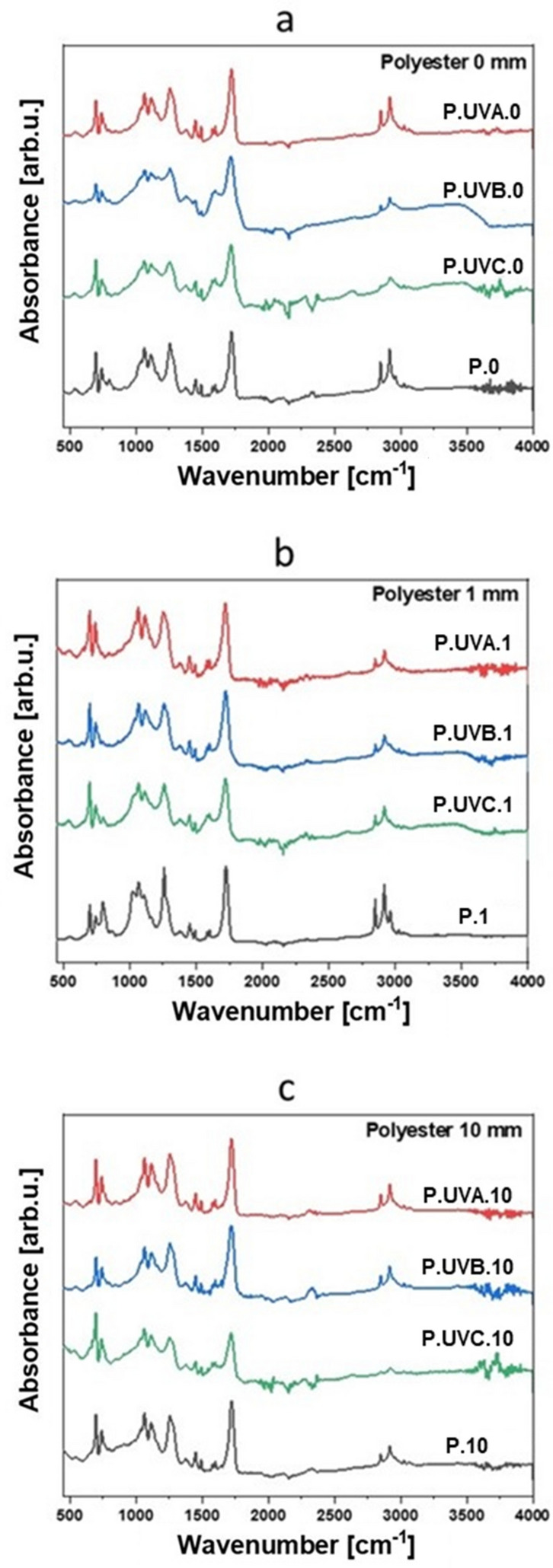
Spectra of FTIR signal for samples not immersed in water (**a**), immersed in water 1 mm deep (**b**), immersed in water 10 mm deep (**c**).

The results obtained using FTIR spectroscopy are presented in Fig. 8. Four characteristic absorption bands are present in each graph: 1721 cm^−1^ (stretching vibration of the C=O group derived from the ester), 1259 cm^−1^ (C(O) –O–C, vibration of ester bond), 1064 cm^−1^ (vibration of C(O)–O–C bond) and 741 cm^−1^ (vibration of C–H bonds in position 1 and 3 in the aromatic ring).

The chemical structure of the cross-linked polyester resin consists of four structural units, i.e. a unit derived from 1,3-propanediol, maleic acid, orthophthalic acid and styrene. The relative content of each of the listed structural units changes as a result of photodegradation. Styrene is subjected to an additional cross-linking process. As a result of hydrolysis, the ester group should be transformed into a carboxyl group, and this reaction is also accompanied by the formation of compounds containing hydroxyl groups. Due to the formation of low-molecular compounds on the surface of the laminate, which dissolve in water, the FTIR spectra do not show bands of stretching vibrations originating from O–H bonds^33,49,50^.

Based on the literature^33,50^, it can be concluded that the cross-linked polyester resin is characterized by a lack of photochemical stability due to the presence of peroxides in the photolysable polystyrene chains. The carbonyl groups formed in the first stages of UV degradation are sensitive to light, which leads to self-acceleration of the destruction reaction as a result of the Norrish type I reaction^49,50^. The reactions result in the formation of quinone and diquinone groups, and the end groups of radicals resulting from the cleavage of the polyester chain and the photolysis of peroxide groups undergo further reactions of radical addition to newly formed quinine groups and oxygen addition to diquinone groups. Eventually, the changes that occur within the composites are manifested by yellowing in color and differentiation of the mechanical properties.

It was found that the number of carbonyl groups increases in the overall balance due to free radical reactions taking place in the resin. The hydrolysis reactions do not occur in an intensified manner during 1000 h, and most of the effects observed in the FTIR spectra result from photochemical changes in the material. Oxidative photodegradation leads to partial disintegration of polymeric polyester chains, resulting in the formation of free radicals, low-molecular products soluble in water, and CO and CO_2_ molecules. Photooxidation of unsaturated polyester initially leads to the formation of quinone and diquinone groups, which is manifested by an increase in the amount of carbonyl groups. The cage effect consisting in the recombination of the formed free radicals or their reaction with the neighboring group is more pronounced in the aquatic environment due to the limited availability of oxygen, which determines the diffusion processes facilitating photooxidation. Free radicals that underwent recombination could lead to the elongation of linear polymer chains or the formation of branched and cross-linked polymer structures. The synergy of phenomena related to the coexistence of the water environment and UV radiation led to the cross-linking of the polyester structure with the participation of free styrene left over from the composite production process, and the free radicals arisen as a result of the cage effect led to the formation of branches in the structure, which caused an increase in the mechanical performance of these laminates. The photodegradation processes taking place are observed on the irradiated side of the composite samples, which has undergone significant destruction, exposing the fibers. The surface character of the polymer composite destruction proves that photooxidation processes are most intense in a thin, near-surface layer. In addition, the reinforcement material is a barrier to UV radiation, which protects the deeper layers of the composite. Low molecular weight compounds formed as a result of photodegradation effectively dissolve in water, which is evidenced by a greater loss of matrix material on samples immersed in distilled water than on samples aged in dry conditions. Samples immersed in water and subjected to UV radiation after the test showed higher mechanical strength, a smaller amount of carbonyl groups and were devoid of the characteristic smell of styrene. In future, the underwater UV exposition tests should be repeated for other types of resins—primarily epoxy and epoxy after the autoclave process. After analyzing an extensive database of results, it will be possible to determine which types of resins are significantly influenced by post-hardening caused by UV rays and the possible synergy of this effect with the presence of water.

## Conclusions

The conducted research allows to conclude that:

- The conducted tests describe the photodegradation of typical GFRP laminates, which are used in many sectors of polymer composites application, are important for the durability and safety of composites in civil, water, air and space engineering,
- Depending on the type of ultraviolet radiation, various changes occur in the GFRP laminate, which is related to the radiation energy: UV-A radiation leads to minor changes, however UV-B and UV-C radiation is strongly destructive to the surface of the composite. Color change and exposure of glass fibers was observed, which is dangerous for exploitation,
- Exposure of the sample to ultraviolet radiation for 1000h causes destruction of the surface and leads to post-curing of the resin in the entire volume, as evidenced by the increase in bending strength. The largest increase was recorded for the UV-A sample immersed in 1 mm and the UV-C sample immersed in 10 mm of water, where there was a change from 240 MPa (reference sample) to about 304 MPa (a change of roughly 30%). Post-curing is also evidenced by the lack of the characteristic smell of styrene in the samples after photoaging,
- The samples immersed in water are characterized by a greater increase in bending strength than the non-immersed samples, which results from the cage effect in which free radicals are trapped in the laminate structure and recombined, and then incorporated into free polymer chains. The post-curing thesis is confirmed by the results obtained by FTIR spectroscopy,
- Immersion of samples in water under ultraviolet radiation allows for a synergistic effect, due to the occurring free radical reactions and the accompanying cage effect causing additional cross-linking of the polyester resin structure, which has a beneficial effect on the mechanical strength of the composite. However, ultraviolet radiation leads to the exposure of fibers on the surface of the composite, which is dangerous in use and excludes the product from operation,
- The proposed “non-normative test method” gives a wider spectrum of possibilities to assess photodegradation processes, also in permanent underwater immersion at adjustable depth.

## Data Availability

The datasets used and/or analysed during the current study available from the corresponding author on reasonable request.
